# Impact of Late ARNI Initiation on Quality of Life and Functional Capacity in CRT-Treated HFrEF Patients: A Single-Centre Cohort Study

**DOI:** 10.3390/jcm15041617

**Published:** 2026-02-19

**Authors:** Oana Patru, Silvia Luca, Dragos Cozma, Cristina Vacarescu, Simina Crisan, Andreea Bena, Mirela Virtosu, Adrian Sebastian Zus, Constantin Tudor Luca, Simona Ruxanda Dragan

**Affiliations:** 1 Cardiology Department, “Victor Babes” University of Medicine and Pharmacy, 2 Eftimie Murgu Sq., 300041 Timisoara, Romania; oana.patru@umft.ro (O.P.); silvia.luca@umft.ro (S.L.); dragos.cozma@umft.ro (D.C.); cristina.vacarescu@umft.ro (C.V.); adrian.zus@umft.ro (A.S.Z.); constantin.luca@umft.ro (C.T.L.); simona.dragan@umft.ro (S.R.D.); 2 Doctoral School, “Victor Babes” University of Medicine and Pharmacy, 300041 Timisoara, Romania; daniela.cozma@umft.ro; 3 Research Center of the Institute of Cardiovascular Diseases Timisoara, 13A Gheorghe Adam Street, 300310 Timisoara, Romania; 4 Institute of Cardiovascular Diseases Timisoara, 13A Gheorghe Adam Street, 300310 Timisoara, Romania; 5 Discipline of Endocrinology, Second Department of Internal Medicine, “Victor Babes” University of Medicine and Pharmacy, 2 Eftimie Murgu Sq., 300041 Timisoara, Romania; bena.andreea@umft.ro; 6 Center for Molecular Research in Nephrology and Vascular Disease, “Victor Babes” University of Medicine and Pharmacy, 2 Eftimie Murgu Sq., 300041 Timisoara, Romania

**Keywords:** heart failure with reduced ejection fraction, cardiac resynchronization therapy, sacubitril/valsartan, quality of life, exercise capacity, reverse remodelling, loop diuretics, delayed therapy initiation, arrhythmic burden, guideline-directed medical therapy

## Abstract

**Background/Objectives:** Cardiac resynchronization therapy (CRT) is a cornerstone treatment for heart failure with reduced ejection fraction (HFrEF), yet many patients remain symptomatic despite long-term electrical optimization. Although sacubitril/valsartan (ARNI) is central to guideline-directed medical therapy (GDMT), data on its late initiation in patients with chronic CRT are scarce. This study evaluated the impact of delayed ARNI initiation on clinical status, functional capacity, and cardiac remodelling in a real-world CRT population. **Methods:** We performed a single-centre, retrospective observational study including 76 HFrEF patients with chronic CRT who started ARNI between 2022 and late 2024. Patients underwent standardized assessment at baseline (T0) and after 12 ± 3 months (T1), including clinical evaluation, 12-item Kansas City Cardiomyopathy Questionnaire (KCCQ-12), symptom-limited bicycle exercise testing, and comprehensive echocardiography. The primary endpoint was change in quality of life (QoL). Secondary endpoints included exercise capacity, echocardiographic reverse remodelling, NYHA class, loop diuretic dose, and device-detected arrhythmias. Dose–response and multidimensional response patterns were explored. **Results:** KCCQ-12 increased from 52.96 ± 16.33 to 75.55 ± 18.12 (Δ +22.59 ± 13.22, *p* < 0.001), with 89.5% achieving a clinically meaningful improvement. Exercise duration and peak workload improved significantly. LVEF increased from 35.08 ± 6.96% to 43.18 ± 8.42% (Δ +8.11%, *p* < 0.001), with reductions in left ventricular and atrial volumes. Loop diuretic dose decreased (median −10 mg/day furosemide equivalent, *p* < 0.001), and 26.3% discontinued diuretics. A lower prevalence of device-detected arrhythmias was observed at follow-up, from 34.2% to 6.6% (*p* < 0.001). Higher ARNI doses were associated with greater likelihood of clinical, functional, and structural response. Longer CRT duration reduced the probability of structural remodelling but not symptomatic or functional benefit. **Conclusions:** In patients with long-standing CRT, delayed ARNI initiation was associated with improvements in QoL, exercise capacity, cardiac remodelling, congestion status, and electrical stability. These findings suggest that CRT is not a therapeutic ceiling and that late ARNI initiation remains a valuable component of comprehensive GDMT.

## 1. Introduction

HFrEF remains a major public health burden, associated with high morbidity, impaired functional capacity, reduced QoL and increased mortality despite substantial therapeutic progress over the past decade [1,2,3]. Cardiac resynchronization therapy (CRT) has become a cornerstone intervention in patients with electrical dyssynchrony, improving left ventricular ejection fraction (LVEF), functional status, exercise capacity and survival [4,5,6]. Nevertheless, even after successful resynchronization, a significant proportion of CRT-treated patients continue to experience symptoms, limitations in daily life and recurrent HF hospitalizations, highlighting the persistent unmet need for further therapeutic optimization [7,8].

ARNI has emerged as a fundamental pharmacological therapy in HFrEF, demonstrating clear superiority over ACE inhibitors in reducing cardiovascular mortality and HF hospitalization [9,10,11]. Although its benefits are well established across a broad spectrum of patients, evidence specifically addressing ARNI initiation in individuals already treated with CRT remains limited, particularly regarding the optimal timing of introduction relative to device therapy. Most available studies evaluate ARNI in mixed HFrEF populations and do not focus on the distinct physiological substrate of chronically resynchronized patients. In addition, in contemporary clinical practice, the majority of HFrEF patients receive SGLT2 inhibitors, which may interact with other neurohormonal therapies and act as potential confounders when assessing the incremental effects of ARNI [12].

Functional capacity and QoL have become essential patient-centred endpoints in HF management, reflecting daily functioning and perceived well-being beyond traditional clinical or echocardiographic parameters. Improvements in QoL correlate strongly with lower hospitalization rates, improved adherence and overall prognosis. Despite this, very few studies have examined QoL evolution specifically in CRT patients following late ARNI initiation, creating a significant knowledge gap in contemporary HF practice [13,14,15,16].

The available evidence suggests that exercise physiology plays a central role in evaluating CRT patients. Cycle ergometer testing has been used to optimize device programming and provides an objective assessment of functional capacity and chronotropic response [17,18], supporting its use for longitudinal evaluation after ARNI initiation. Furthermore, recent analyses underline the need for refined patient selection criteria for CRT fusion pacing [19,20,21], emphasizing the role of advanced imaging markers such as mitral annular plane systolic excursion (MAPSE) in understanding residual systolic dysfunction [22,23]. Together, these findings highlight a multifaceted landscape in which CRT-treated patients may benefit from additional pharmacological interventions targeting persistent symptoms and incomplete reverse remodelling.

Our centre systematically introduced ARNI therapy in patients with long-standing CRT following the expansion of the national reimbursement protocol in 2022, when sacubitril/valsartan became widely accessible in Romania. Before this change, its high cost had markedly limited its use, particularly in patients already treated with device therapy such as CRT. This context created a unique real-world cohort of previously resynchronized patients who were systematically hospitalized for ARNI initiation, allowing standardized baseline clinical, functional and echocardiographic assessment (T0), including echocardiography, a symptom-limited bicycle exercise test and a validated QoL questionnaire, followed by guided dose titration to the maximum tolerated levels and re-evaluation at approximately 12 months (T1).

This design enables evaluation of the incremental benefit of delayed ARNI therapy in a population already optimized through CRT and previously limited by restricted access to this treatment. Although both CRT and ARNI are established therapies in HFrEF, the clinical and functional impact of initiating ARNI several years after device implantation remains insufficiently defined. We therefore hypothesized that late ARNI introduction may still yield clinically meaningful improvements in patient-reported outcomes, exercise capacity, and cardiac remodelling, and aimed to provide real-world data on multidimensional clinical, functional, and structural outcomes in patients with long-standing CRT.

## 2. Materials and Methods

This was a retrospective, observational cohort study conducted in a single tertiary cardiac center with an established HF and device therapy program. The study followed a single-arm observational design without a parallel control or comparator group. We included consecutive patients with HFrEF and CRT in whom ARNI therapy was initiated between 2022 and late 2024, following national reimbursement approval that allowed systematic ARNI prescription in patients already optimized on CRT.

All clinical, laboratory, echocardiographic, and functional data were extracted from electronic medical records at two predefined time points: baseline (T0), corresponding to the hospitalization for ARNI initiation, and follow-up (T1), corresponding to a routine follow-up visit performed 12 ± 3 months after ARNI introduction.

### 2.1. Patient Selection

Eligible patients were adults (≥18 years) with established HFrEF (left ventricular ejection fraction [LVEF] ≤ 40%) who had undergone CRT-P or CRT-D implantation at least 12 months prior to ARNI initiation and were clinically stable on GDMT. All patients fulfilled national criteria for ARNI prescription, were able to perform a symptom-limited bicycle exercise test, and were able to complete a validated QoL questionnaire.

Patients were excluded if they presented with acute decompensated HF at baseline, had significant renal dysfunction (estimated glomerular filtration rate <30 mL/min/1.73 m^2^), symptomatic hypotension precluding ARNI initiation, recent myocardial infarction (<3 months), severe valvular disease requiring intervention, or were unable to complete exercise testing or QoL assessment. Only patients who successfully initiated and tolerated ARNI therapy were included in the analysis. This approach may introduce a degree of selection bias toward clinically stable individuals with higher treatment tolerance.

### 2.2. Baseline Characteristics

At baseline, demographic and clinical data included age, sex, HF etiology (ischemic vs. non-ischemic), and relevant comorbidities (atrial fibrillation, ventricular tachycardia). Device-related data comprised CRT type (CRT-P or CRT-D) and CRT duration prior to ARNI initiation, expressed in years. CRT duration was recorded as a potential modifier of therapeutic response, given that reverse remodelling after CRT evolves over time and baseline myocardial remodelling stage may influence the incremental benefit observed after ARNI introduction. Background GDMT was documented for all patients. At baseline, all patients were receiving maximally tolerated GDMT, including beta-blockers (beta-blocker therapy was already optimized to the maximum tolerated dose with the dual aim of optimizing HF therapy and achieving the highest possible percentage of effective biventricular pacing) and mineralocorticoid receptor antagonists when tolerated. In addition, 60 of 76 patients (79%) were already treated with an SGLT2 inhibitor. In this context, ARNI initiation represented the main structured therapeutic escalation during follow-up. No other major changes in background HF therapy were systematically introduced during the follow-up period.

### 2.3. ARNI Initiation and Titration Protocol

Sacubitril/valsartan was initiated during a dedicated hospital admission at baseline (T0), according to a standardized protocol adapted from ESC/HFA recommendations [24] and national reimbursement regulations. The initial dose (24/26 mg or 49/51 mg twice daily) was selected based on blood pressure, renal function, serum potassium levels, and prior ACE inhibitor or ARB therapy.

For analytical purposes, doses were expressed as daily sacubitril/valsartan equivalents: 100 mg/day for 24/26 mg twice daily, 200 mg/day for 49/51 mg twice daily, and 400 mg/day for 97/103 mg twice daily. Up-titration toward the maximum tolerated dose was performed in outpatient care at 4–8 weeks intervals. The final tolerated dose was recorded at follow-up (T1).

### 2.4. Clinical and Functional Assessment

At both baseline and follow-up, patients underwent standardized clinical evaluation, including assessment of NYHA functional class, blood pressure, heart rate, and body weight. Daily loop diuretic dose, expressed as furosemide equivalents, was recorded. Device interrogation was used to document the presence of atrial or ventricular arrhythmias during the 12 months preceding baseline and during the follow-up period between T0 and T1.

### 2.5. QoL Assessment

QoL was evaluated using the 12-item Kansas City Cardiomyopathy Questionnaire (KCCQ-12). Patients completed the questionnaire at baseline and again at follow-up. Raw scores ranged from 12 to 60 and were transformed into standardized scores from 0 to 100 using the formula: KCCQ score = (Raw score − 12)/48 × 100.

Higher scores indicated better health status. Clinically meaningful changes were defined as an increase of at least 5 points (minimal important difference), at least 10 points (moderate improvement), and at least 20 points (large improvement).

### 2.6. Exercise Testing

Functional capacity was assessed using a symptom-limited bicycle exercise test rather than the 6-min walk test. Cycle ergometry was chosen because it allows controlled and reproducible workload increments, provides direct measurement of mechanical output, and enables continuous ECG and blood pressure monitoring, which is particularly relevant in CRT recipients with advanced HF. Compared with walking-based tests, bicycle ergometry is less affected by gait instability, frailty, or musculoskeletal limitations and provides a more standardized evaluation of exercise tolerance and chronotropic response. For these reasons, symptom-limited bicycle ergometry represents a suitable and physiologically meaningful instrument for detecting functional changes after ARNI initiation, offering greater resolution in assessing treatment response while maintaining feasibility and real-world applicability [18].

All tests were performed on an electronically braked cycle ergometer under continuous ECG and blood pressure monitoring. At both baseline and follow-up, patients started at a low initial workload (typically 25 W), followed by stepwise increases of 25 W every 90 s, adjusted to clinical tolerance. Exercise was continued until symptom limitation (fatigue, dyspnea, or angina), development of significant arrhythmias, abnormal blood pressure response, or physician decision to terminate the test.

Total exercise duration (in minutes), peak workload (in watts), peak heart rate, and symptoms at termination were recorded. Peak workload expressed in watts was used as the primary measure of exercise capacity, as it represents a direct device-derived mechanical output, whereas metabolic equivalents require formula-based conversion and may introduce additional variability, particularly in CRT patients receiving beta-blockers or heart rate-lowering therapy [25].

### 2.7. Echocardiographic Evaluation

Comprehensive transthoracic echocardiography was performed at baseline and follow-up by experienced cardiologists who were blinded to QoL data. LVEF was measured using the biplane Simpson method. Left ventricular end-diastolic volume (LVEDV), left ventricular end-systolic volume (LVESV), and left atrial volume (LAV) were also recorded.

Historical LVEF values prior to CRT implantation were collected for descriptive purposes, but were not included in the primary analysis, as the study focused on the incremental effect of ARNI on top of established CRT. Reverse remodelling was defined as a relative reduction of at least 10% in LVESV or an absolute increase of at least 5% in LVEF.

### 2.8. Diuretic Use and Arrhythmia Burden

Daily loop diuretic dose, expressed as furosemide equivalents, was recorded at both baseline and follow-up. Use of spironolactone was documented but was not considered a marker of congestion. Arrhythmia burden was defined by the presence of non-sustained ventricular tachycardia, atrial fibrillation episodes, and appropriate implantable cardioverter-defibrillator therapies during the 12 months before baseline and during the follow-up interval between T0 and T1. All CRT devices were interrogated systematically at each visit.

### 2.9. Outcomes

The primary outcome was the change in KCCQ-12 overall summary score from baseline to follow-up. Secondary outcomes included changes in exercise capacity (exercise duration and peak workload), echocardiographic parameters of reverse remodelling (LVEF, LVEDV, LVESV, and LAV), NYHA functional class, and daily loop diuretic dose. In addition, patients were classified according to response to therapy using predefined clinically meaningful thresholds across three domains: QoL, functional capacity, and cardiac remodelling.

A QoL responder was defined as a patient with an increase of at least 10 points in the KCCQ-12 overall summary score between baseline and follow-up. A functional (exercise) responder was defined as a patient with an increase in exercise capacity of at least 25 W in peak workload or an increase of at least 1 min in total exercise duration. An echocardiographic responder was defined as a patient with an absolute increase in LVEF of at least 5% at follow-up.

Based on these criteria, patients were further categorized into response patterns. Patients meeting all three response criteria were defined as global responders. Patients showing improvement in quality of life without concomitant echocardiographic response were considered to have a predominantly symptomatic response pattern. Conversely, patients exhibiting echocardiographic reverse remodelling in the absence of significant symptomatic or functional improvement were classified as having a delayed or dissociated response pattern. The use of multidimensional responder definitions was intended to reflect the heterogeneous clinical expression of treatment benefit in HFrEF, where symptomatic, functional, and structural improvements do not always occur simultaneously.

### 2.10. Statistical Analysis

Continuous variables are presented as mean ± standard deviation or median with interquartile range, as appropriate. Categorical variables are expressed as absolute counts and percentages. The distribution of continuous variables was assessed using the Shapiro–Wilk test.

Paired comparisons between baseline (T0) and follow-up (T1) were performed using parametric or non-parametric tests according to data distribution. For continuous variables, paired-samples Student’s t-test was used when within-subject differences were normally distributed, while the Wilcoxon signed-rank test was applied otherwise. Paired binary outcomes were compared using McNemar’s exact test. Proportions were additionally analyzed using two-sided exact binomial tests when appropriate. Correlations between continuous or ordinal variables were assessed using Spearman’s rank correlation coefficient (ρ). To account for multiple comparisons, *p*-values were adjusted using the Benjamini–Hochberg false discovery rate (FDR) procedure, and FDR-adjusted q-values are reported where relevant. To address potential confounding by baseline characteristics, we performed multivariable regression analyses. For continuous outcomes (change in LVEF and change in KCCQ-12 score), we constructed linear regression models using the change from baseline to follow-up (Δ = T1 − T0) as the dependent variable. Each model included age, sex, baseline NYHA class, cardiomyopathy etiology (ischemic vs. non-ischemic), and CRT device type as covariates. Regression coefficients, confidence intervals and *p*-values were examined to determine independent associations. Given the modest sample size, results of multivariable regression analyses should be interpreted with caution. These models were introduced to provide additional adjustment and were not intended to establish causal inference. All statistical tests were two-sided, and a *p* value < 0.05 was considered statistically significant. All analyses were performed using MedCalc Statistical Software version 12.5 (MedCalc Software Ltd., Ostend, Belgium). No relevant missing data were identified for the primary or secondary endpoints.

The study protocol was approved by the local institutional ethics committees, and all procedures complied with the Declaration of Helsinki. Due to the retrospective design and use of routinely collected data, no additional informed consent was required beyond standard hospital admission consent.

## 3. Results

### 3.1. Study Population and ARNI Exposure

A total of 76 patients with HFrEF and chronic CRT treated with ARNI were included in the analysis. All patients began ARNI therapy between 2022 and 2024, coinciding with national reimbursement approval. The cohort consisted predominantly of older patients (mean age 67.6 ± 10.99 years), with a male predominance (68.4%). Cardiomyopathy etiology was non-ischemic in 68.4% and ischemic in 31.6% of cases. At baseline, all patients were in NYHA class II or III, with a mean LVEF of 35.08 ± 6.96%. Median duration of CRT before ARNI initiation was 5.5 [4.0–7.0] years, reflecting a population with long-standing device therapy prior to pharmacological escalation.

Regarding ARNI dose distribution at follow-up, 39.5% of patients (*n* = 30) were maintained on 100 mg/day, 44.7% (*n* = 34) on 200 mg/day, and 15.8% (*n* = 12) achieved the maximum dose of 400 mg/day. Baseline demographic and clinical characteristics are summarized in Table 1.

### 3.2. Clinical and Functional Response to ARNI

The dynamic changes in QoL, exercise performance, and echocardiographic parameters from baseline to follow-up are summarized in Table 2.

QoL, assessed by the KCCQ-12 overall summary score, improved markedly after ARNI initiation, with a mean absolute increase of +22.59 ± 13.22 points (95% CI, 19.62 − 25.56; *p* < 0.001). A clinically meaningful improvement was observed in the vast majority of patients, with 89.5% achieving an increase of at least 10 points, indicating a substantial patient-perceived benefit following ARNI introduction (Table 2).

Exercise performance on symptom-limited bicycle ergometry improved significantly during follow-up. Both exercise duration and peak workload increased, demonstrating a consistent improvement in objective functional capacity after ARNI initiation (all *p* < 0.001; Table 2).

**Figure 1:**
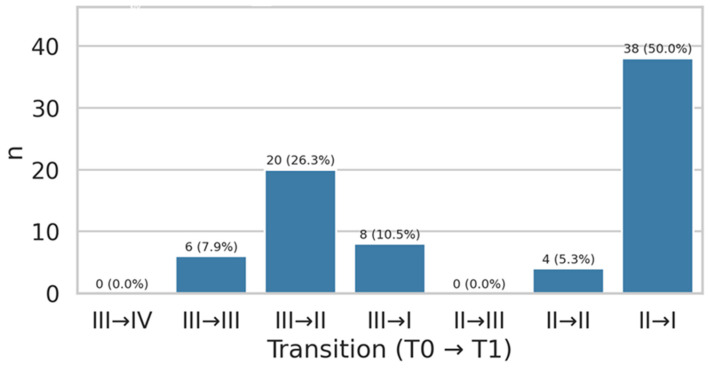
NYHA class transitions from baseline (T0) to follow-up (T1) in the study population.

Between baseline and follow-up, NYHA class improved in 66 of 76 patients (86.8%), remained unchanged in 10 patients (13.2%), and worsened in none. The most frequent transition was from NYHA class II to I (38/76, 50.0%), followed by class III to II (20/76, 26.3%). Overall, these data confirm a substantial improvement in clinician-assessed functional status after ARNI initiation, as seen in Figure 1.

### 3.3. Cardiac Remodelling and Congestion

Significant additional reverse remodelling was observed despite long-standing CRT. LVEF increased from 35.08 ± 6.96% at baseline to 43.18 ± 8.42% at follow-up, with a mean absolute increase of 8.11 ± 5.74% (95% CI 6.82–9.40, *p* < 0.001). Left ventricular end-diastolic volume decreased from 211.79 ± 86.86 mL to 177.58 ± 74.74 mL (Δ −34.21 ± 46.34 mL, *p* < 0.001). Left atrial volume also decreased significantly, from 92.63 ± 30.43 mL to 80.87 ± 28.21 mL (Δ −11.76 ± 26.81 mL, *p* < 0.001) (Table 2).

These findings indicate that ARNI therapy provided an incremental structural benefit on top of established CRT-mediated remodelling.

Loop diuretic dose decreased significantly after ARNI initiation (paired Wilcoxon signed-rank test, *p* < 0.001), with a median change of −10 mg/day of furosemide-equivalent.

**Figure 2:**
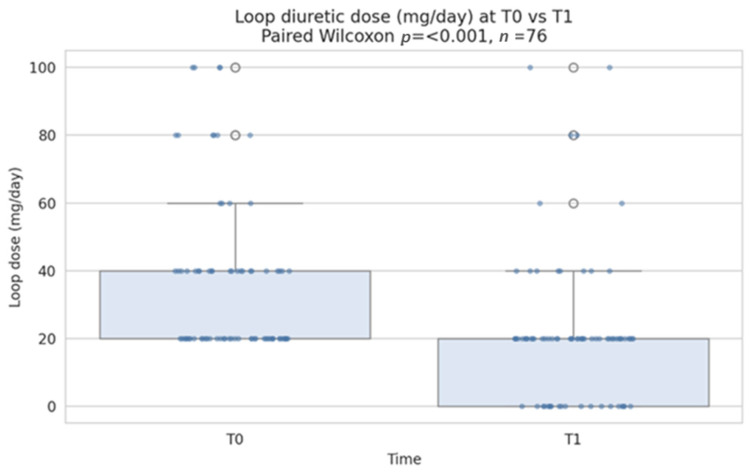
Distribution plot of loop dose at baseline vs. follow-up (T0 = baseline; T1 = follow-up). Dots represent individual patient values.

Overall, 50% of patients required a reduction in loop diuretic dose, among whom 26.3% were able to discontinue loop diuretics completely. The remaining 50% maintained a stable dose, and none required dose escalation during follow-up. These findings suggest an improvement in congestion status and hemodynamic stability after ARNI therapy. A distribution plot of loop diuretic dose at baseline vs. follow-up (boxplot + individual points, annotated with the paired *p*-value) is displayed in Figure 2.

### 3.4. Arrhythmic Burden

The prevalence of device-detected arrhythmias decreased significantly after ARNI initiation. Any arrhythmia was present in 26 patients (34.2%) at baseline and in only 5 patients (6.6%) at follow-up (*p* < 0.001). At baseline, atrial fibrillation was documented in 22 patients (28.9%) and non-sustained ventricular tachycardia in 6 patients (7.9%), with 2 patients presenting both arrhythmia types. At follow-up, atrial fibrillation episodes were observed in 5 patients (6.6%), while no ventricular tachycardia episodes were recorded (*p* = 0.031). No new-onset arrhythmias were observed during the follow-up period. Overall, these findings indicate a decrease in arrhythmic burden coinciding with ARNI initiation. Consistent with the reduction in arrhythmic burden, no appropriate ICD therapies (anti-tachycardia pacing or shocks) were recorded during the follow-up period. Arrhythmic data reflect the prevalence of device-detected episodes during the observation intervals rather than standardized incidence rates.

### 3.5. Multidimensional Treatment Response and Determinants

#### 3.5.1. Responder Analysis

Table 3 summarizes the distribution of responders across the predefined response domains. A high proportion of patients fulfilled responder criteria in each individual category, with the largest proportion observed for QoL and echocardiographic response. More than half of the cohort met criteria across all three domains and were classified as global responders. This distribution highlights that symptomatic, functional, and structural responses were frequently concordant, but not universal, supporting the concept of a multidimensional and heterogeneous treatment response to ARNI therapy. These multidimensional response patterns may provide a pragmatic framework for individualized therapeutic evaluation in routine clinical practice.

#### 3.5.2. Dose–Response Relationship

A consistent dose–response relationship was observed between ARNI dose and treatment response across all predefined domains. Higher ARNI doses were associated with a greater likelihood of being classified as a responder for QoL, functional capacity, echocardiographic remodelling, and global response. Specifically, ARNI dose was positively correlated with QoL response (*ρ* = 0.255, q = 0.035), functional response (*ρ* = 0.236, q = 0.040), echocardiographic response (*ρ* = 0.265, q = 0.035), and global response (*ρ* = 0.323, q = 0.018).

**Figure 3:**
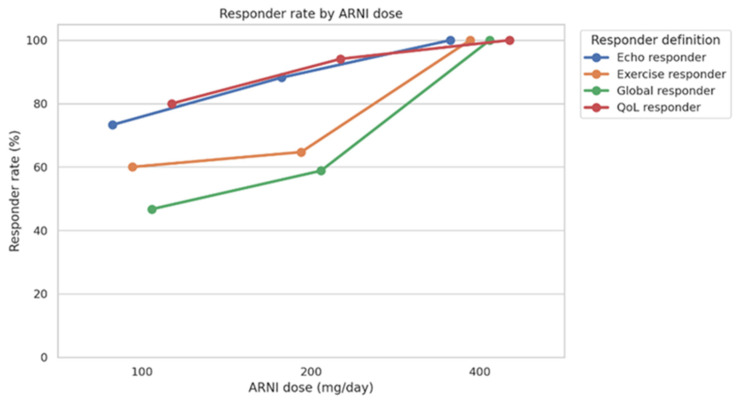
Responder rates across ARNI dose categories. Responder rates for QoL, exercise capacity, echocardiographic response, and global response are shown across ARNI dose categories (100, 200, and 400 mg/day). Each colored line represents a distinct response domain. Dots indicate the proportion of responders in each dose group, with connecting lines used to facilitate visualization of trends. Percentages are calculated within each dose category.

Responder rates increased progressively across dose categories (100, 200, and 400 mg/day), with the highest proportions consistently observed in patients receiving the maximum ARNI dose, as seen in Figure 3. The dose-associated pattern was most pronounced for global responders, supporting a cumulative effect of ARNI dose on multidimensional clinical response.

#### 3.5.3. Impact of Cardiomyopathy Etiology

**Figure 4:**
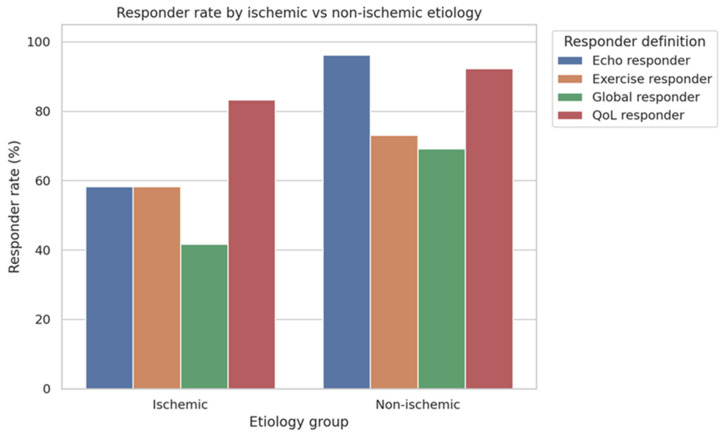
Responder rates according to cardiomyopathy etiology. Responder rates for QoL, exercise capacity, echocardiographic response, and global response stratified by ischemic and non-ischemic etiology. Non-ischemic patients exhibited higher responder rates, particularly for echocardiographic and QoL response domains. Bars represent the percentage of responders within each etiological group. This figure is descriptive and intended for exploratory visualization.

Ischemic etiology was not significantly associated with QoL or exercise (functional) response. However, ischemic cardiomyopathy was negatively associated with echocardiographic (*p* = −0.482, q < 0.001) and global response (*p* = −0.262, q = 0.044), indicating a higher probability of structural reverse remodelling and multidimensional response in patients with non-ischemic cardiomyopathy in this population, after ARNI initiation, as shown in Figure 4.

An additional exploratory visualization of responder distributions simultaneously stratified by ARNI dose and cardiomyopathy etiology is provided in Figure S1 (Supplementary Materials).

#### 3.5.4. CRT Duration and Probability of Response (Exploratory Analysis)

**Figure 5:**
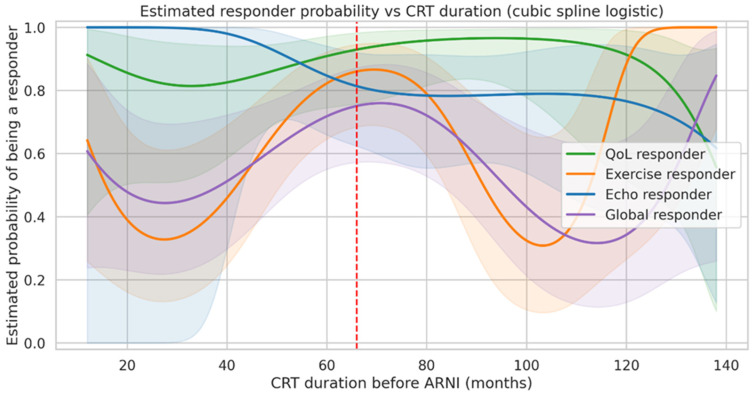
Estimated probability of treatment response according to CRT duration. The probability of being classified as a responder is shown as a function of CRT duration prior to ARNI initiation, modeled using logistic regression with cubic spline functions. Separate curves are displayed for QoL, exercise, echocardiographic, and global response. Shaded areas represent 95% confidence intervals. The dashed vertical line indicates the approximate transition zone around 66 months (5.5 years). Predictions are displayed only within the range of CRT duration supported by the observed data.

The relationship between CRT duration prior to ARNI initiation and probability of treatment response was explored using logistic regression models with cubic spline functions as illustrated in Figure 5.

For echocardiographic response, the modeled probability decreased with increasing CRT duration, with a visible transition zone around 5.5–6 years (66–72 months), suggesting that patients with long-standing CRT may have a reduced potential for further structural remodelling.

For global response, the modeled relationship was non-linear and characterized by wider confidence intervals, indicating increased uncertainty and a less robust association. Exercise response probability showed only minor variation across CRT durations, while QoL response remained high across the entire range of CRT exposure. These findings suggest that the duration of prior CRT may modulate structural but not necessarily symptomatic or functional response to ARNI.

A detailed visualization of the echocardiographic response model is provided in Figure S2 (Supplementary Materials).

#### 3.5.5. Baseline Severity and Magnitude of Improvement

**Figure 6:**
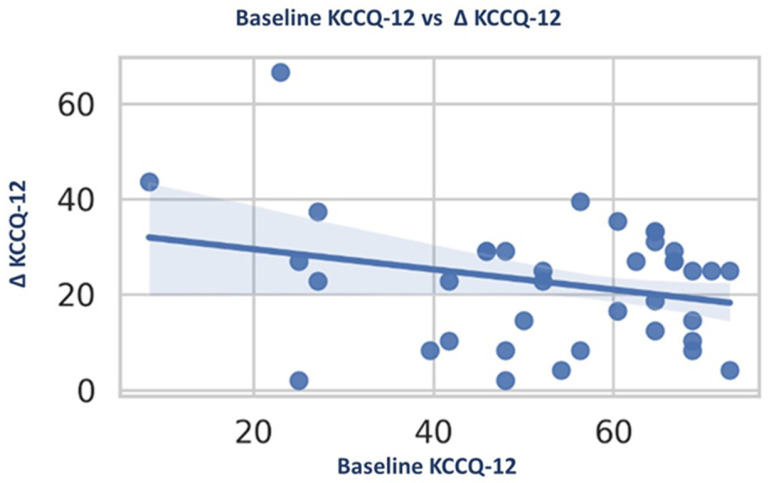
Relationship between baseline KCCQ-12 score and improvement in QoL. Scatter plot showing the association between baseline KCCQ-12 score and change in KCCQ-12 (ΔKCCQ-12) during follow-up. Dots represent individual observations. Lower baseline KCCQ-12 scores were associated with greater improvement in QoL. The solid line represents the fitted linear regression model, with shaded areas indicating the 95% confidence interval.

Baseline patient-reported functional status showed a modest but significant inverse relationship with subsequent improvement in QoL. Lower baseline KCCQ-12 scores were associated with larger absolute gains during follow-up. In linear regression analysis, baseline KCCQ-12 score was independently and inversely associated with ΔKCCQ-12 (β = −0.21 per 1-point increase; 95% CI −0.39 to −0.03; R^2^ = 0.07; *p* = 0.022). This relationship is illustrated in Figure 6.

In contrast, baseline NYHA class and baseline LVEF were not significantly associated with their respective changes, indicating that clinician-assessed functional and echocardiographic improvements were largely independent of baseline disease severity.

#### 3.5.6. Multivariable Regression Analyses

Multivariable linear regression analysis was performed with ΔLVEF as the dependent variable, adjusting for age, sex, baseline NYHA class, cardiomyopathy etiology (ischemic vs. non-ischemic), and CRT device type. The overall model was significant (R^2^ = 0.34, *p* = 0.0002). None of the included baseline variables showed a statistically significant independent association with change in LVEF.

In the multivariable model for change in KCCQ-12 score, the overall model was also significant (R^2^ = 0.36, *p* < 0.001). Older age was associated with a modestly smaller improvement in KCCQ-12, whereas sex, cardiomyopathy etiology, and CRT device type were not significantly associated with change in KCCQ-12 after adjustment.

## 4. Discussion

Patients with long-standing CRT represent a distinct and often underexplored HFrEF population. Although they are electrically optimized, usually display stable mechanical synchrony, and have already undergone a substantial degree of reverse remodelling, they are commonly perceived as having limited potential for further therapeutic gain [26,27]. Our findings suggest that this assumption may not fully apply in all patients. Our results indicate that CRT may not represent a therapeutic ceiling in HFrEF and that significant residual neurohormonal reversibility persists even years after mechanical resynchronization. In this setting, ARNI initiation was associated with clinically meaningful improvements across multiple domains, including QoL, functional capacity, ventricular remodelling, congestion status and arrhythmic burden.

The concordant improvement in patient-reported outcomes, exercise performance and echocardiographic parameters supports a true multidimensional biological effect of ARNI, rather than an isolated or placebo-driven symptomatic response. This coherence across subjective and objective endpoints strengthens the internal validity of our findings and suggests that late ARNI initiation acts through integrated hemodynamic, neurohormonal and myocardial mechanisms, further enhancing myocardial efficiency and unloading even in electrically optimized ventricles. Compared with landmark trials such as PARADIGM-HF [9], which predominantly enrolled patients without device therapy, our cohort reflects a more advanced and therapeutically saturated HF phenotype. The magnitude of QoL improvement observed is therefore particularly striking and argues strongly against therapeutic inertia once CRT has been established.

The significant reduction in loop diuretic requirement further supports the hemodynamic relevance of ARNI in this setting. Decreasing reliance on chronic diuretic therapy suggests improved congestion control and cardiovascular stability. This is clinically meaningful, as prolonged exposure to high-dose loop diuretics has been associated with worsening renal function, neurohormonal activation, electrolyte disturbances and adverse prognosis [28]. Thus, ARNI appears not only to improve symptoms and structure but also to shift patients toward a more favorable long-term therapeutic profile.

Although most patients were receiving SGLT2 inhibitors, these agents were administered at fixed doses and remained stable throughout follow-up, whereas ARNI was the only therapy systematically initiated and up-titrated during the study period. The observed clinical, functional, and echocardiographic improvements therefore likely reflect the incremental contribution of ARNI on top of optimized GDMT rather than parallel pharmacological intensification. Nevertheless, a potential synergistic interaction between ARNI and SGLT2 inhibitors cannot be excluded, as both drug classes exert complementary hemodynamic and neurohormonal effects [9,29]. The consistent improvement also observed in patients not treated with SGLT2 inhibitors further supports an independent contribution of ARNI to congestion relief, functional recovery, and reverse remodelling.

The reduction in arrhythmic burden observed after ARNI initiation adds another layer of clinical relevance. Improved electrical stability may reflect reduced ventricular wall stress, enhanced myocardial energetics and attenuation of neurohormonal activation. These mechanisms are well described for ARNI and could be particularly relevant in a synchronized ventricle, where mechanical efficiency is already optimized by CRT. The absence of appropriate ICD therapies during follow-up is consistent with the hypothesis of a more stable electrophysiological substrate after ARNI initiation [30,31]; however, these findings should be interpreted as hypothesis-generating rather than evidence of a direct antiarrhythmic effect.

The exploratory analysis of CRT duration provides additional pathophysiological insight. While the probability of echocardiographic response decreased with longer CRT exposure, symptomatic and functional benefits remained largely preserved across the entire spectrum of CRT duration. This dissociation suggests that even when structural remodelling potential becomes partially exhausted, meaningful improvements in QoL and exercise tolerance remain achievable. These findings highlight the importance of not equating the absence of further remodelling with therapeutic futility and support ARNI initiation regardless of the time elapsed since device implantation.

The reimbursement-driven introduction of ARNI in Romania created a natural experiment that allowed the evaluation of delayed ARNI initiation in a population that is otherwise underrepresented in randomized trials. Unlike controlled studies that typically enroll patients early in their therapeutic trajectory, our cohort reflects real-life, long-term CRT recipients. This strengthens the external validity and contemporary clinical relevance of our findings, particularly for healthcare systems where ARNI access has expanded only recently.

Overall, our results reinforce the concept that late ARNI initiation remains clinically worthwhile in CRT-treated HFrEF patients. ARNI provides incremental benefit beyond mechanical resynchronization alone, improving patient-centred outcomes, functional performance, cardiac structure, hemodynamic stability and electrical stability. These effects support a comprehensive and persistent role of neurohormonal modulation even in patients already optimized by advanced device therapy.

### 4.1. Future Directions

Future studies should validate these findings in larger, multicentre cohorts and prospectively evaluate QoL, exercise capacity and cardiac remodelling in CRT-treated patients initiating ARNI. Particular attention should be given to CRT duration prior to ARNI initiation, as this parameter may help identify subgroups with different remodelling trajectories and therapeutic responsiveness. Dedicated studies stratifying patients by CRT longevity (e.g., <1 year vs. ≥1 year) could clarify whether ARNI primarily enhances early remodelling or reactivates reverse remodelling in long-standing CRT recipients. The integration of hemodynamic parameters and standardized congestion biomarkers would further refine patient selection and mechanistic understanding.

### 4.2. Limitations

Several important limitations must be acknowledged. First, its retrospective, single-centre, observational design without a control group precludes any causal inference. The observed changes after ARNI initiation can only be interpreted as associations and cannot be definitively attributed to the pharmacological effect of sacubitril/valsartan. Regression to the mean, survivor bias, and the natural evolution of HF under intensified clinical follow-up may all have contributed to the observed improvements. Second, the cohort represents a highly selected population. All patients were stable enough to undergo ARNI initiation, complete exercise testing, and attend long-term follow-up, which may have introduced selection bias toward patients with better baseline prognosis and higher treatment adherence. Patients who did not tolerate ARNI, experienced early adverse events, or were lost to follow-up are not captured, potentially leading to an overestimation of treatment-associated benefits. Third, although most patients were already receiving contemporary GDMT including SGLT2 inhibitors, residual confounding by background therapies cannot be completely excluded. In addition, although exploratory analyses did not suggest a dominant influence of baseline demographic or clinical characteristics such as sex, cardiomyopathy etiology, NYHA class, or CRT device type, residual confounding related to these variables cannot be entirely ruled out. Changes in adherence, lifestyle, or subtle medication adjustments during follow-up may have contributed to the observed effects.

Fourth, the dose–response analyses should be considered exploratory. Higher ARNI doses may reflect better baseline hemodynamic stability, renal function, and overall clinical robustness rather than a true causal relationship between dose and magnitude of response. Dose may, therefore, act as a marker of patient tolerance and clinical reserve rather than as a direct determinant of outcome. Notably, the multidimensional response pattern was most pronounced at the highest ARNI dose, with global and exercise responders being particularly represented in this subgroup, highlighting the potential clinical relevance of progressive dose titration whenever tolerated.

Fifth, the analysis of arrhythmic burden was based on device-detected events and reflects prevalence rather than standardized event rates. Differences in monitoring intensity, detection thresholds, and device programming over time may have influenced arrhythmia reporting. Consequently, these findings should be interpreted as descriptive and hypothesis-generating.

Sixth, the modelling of CRT duration using spline-based regression is limited by the modest sample size and should be regarded as exploratory. These analyses were intended to describe potential trends rather than establish definitive relationships and are subject to overfitting and wide uncertainty intervals.

Finally, the absence of systematic NT-proBNP measurements prevents direct correlation of clinical and echocardiographic findings with objective biomarkers of neurohormonal activation and limits mechanistic interpretation. However, this reflects real-world constraints in many healthcare systems and underscores the pragmatic nature of the study. The consistency between symptomatic improvement, functional capacity and echocardiographic improvements nevertheless provides indirect support for a biologically meaningful effect, even in the absence of biomarker confirmation. Future prospective studies incorporating systematic biomarker assessment may further clarify these mechanisms.

Despite these limitations, the study offers real-world evidence that late initiation of ARNI in CRT-treated HFrEF patients is associated with clinically meaningful improvements across several relevant domains. Although causality cannot be established, the consistency of the observed changes suggests that patients with long-standing CRT may still derive significant benefit from further pharmacological optimization. These findings question the perception that this population is therapeutically exhausted and indicate that a residual potential for clinical, functional and structural improvement may persist even years after device implantation.

## 5. Conclusions

In this real-world cohort of patients with long-standing CRT, delayed initiation of sacubitril/valsartan was primarily associated with improvements in QoL and exercise capacity, representing the most consistent findings of the study. Additional benefits were observed in echocardiographic remodelling and congestion status, while the reduction in device-detected arrhythmias should be considered descriptive and hypothesis-generating. Although the retrospective and uncontrolled design precludes causal inference, these findings suggest that clinically meaningful benefits may still be observed after ARNI initiation even several years after CRT implantation. Overall, CRT should not be regarded as a therapeutic endpoint in HFrEF management. Late ARNI initiation may represent a valuable component of comprehensive GDMT in chronically resynchronized patients who remain symptomatic despite optimized device therapy or exhibit incomplete reverse remodelling; however, these findings require prospective validation before routine clinical implementation.

## Figures and Tables

**Table 1:** Baseline demographic and clinical characteristics of the study population.

Variable	Total Cohort (*n* = 76)
Age, years	67.58 ± 10.99
Sex	Male: 52 (68.4%); Female: 24 (31.6%)
Etiology of cardiomyopathy	Ischemic: 24 (31.6%); Non-ischemic: 52 (68.4%)
NYHA class at baseline	II: 42 (55.3%); III: 34 (44.7%)
LVEF at baseline, %	35.08 ± 6.96
LVEDV, mL	211.79 ± 86.86
LAV, mL	92.63 ± 30.43
CRT duration before ARNI, years	5.5 [4.0–7.0]
Type of CRT device	CRT-D: 32 (42.1%); CRT-P: 44 (57.9%);
HF hospitalizations in the prior 12 months	2.0 [2.0, 3.0]
Pre-ARNI loop diuretic dose, mg/day (furosemide equivalent)	36.84 ± 23.34
Baseline KCCQ-12 score (0–100)	52.96 ± 16.33
Baseline exercise test duration, minutes	5.46 ± 1.77
Baseline exercise test peak workload, Watts	103.95 ± 31.37
Any device-detected arrhythmia at baseline	26 (34.2%) (AF: 22 (28.9%); VT: 6 (7.9%), with 2 patients having both)

**Table 2:** Dynamic changes in QoL, functional capacity, and echocardiographic parameters from baseline to follow-up.

Outcome	Baseline (T0)	Follow-Up (T1)	Δ (T1–T0)	*p*-Value
**Quality of life & functional capacity**				
KCCQ-12 score (0–100)	52.96 ± 16.33	75.55 ± 18.12	+22.59 ± 13.22	<0.001
Bicycle test duration (minutes)	5.46 ± 1.77	6.85 ± 2.05	+1.40 ± 1.12	<0.001
Peak workload (Watts)	103.95 ± 31.37	124.34 ± 38.51	+20.39 ± 25.07	<0.001
**Echocardiographic parameters**				
LVEF (%)	35.08 ± 6.96	43.18 ± 8.42	+8.11 ± 5.74	<0.001
LV end-diastolic volume (mL)	211.79 ± 86.86	177.58 ± 74.74	−34.21 ± 46.34	<0.001
Left atrial volume (mL)	92.63 ± 30.43	80.87 ± 28.21	−11.76 ± 26.81	<0.001

Data are presented as mean ± standard deviation; T0 = baseline evaluation; T1 = follow-up evaluation at 12 ± 3 months; Δ = change between T1 and T0.

**Table 3:** Distribution of responders across predefined response domains.

Response Category	Definition	*n* (%)
QoL responders	ΔKCCQ-12 ≥ 10 points	68 (89.5%)
Functional responders	ΔPeak workload ≥ 25 W and/or ΔExercise duration ≥ 1 min	52 (68.4%)
Echocardiographic responders	ΔLVEF ≥ 5% (absolute increase)	64 (84.2%)
Global responders	Fulfilled criteria for QoL, functional, and echocardiographic response	46 (60.5%)

## Data Availability

The original contributions presented in this study are included in the article. Further inquiries can be directed to the corresponding author.

## Supplementary Materials

The following supporting information can be downloaded at: https://www.mdpi.com/article/10.3390/jcm15041617/s1, Figure S1. Responder rate heatmaps stratified by ARNI dose and cardiomyopathy etiology. Heatmaps display the proportion of responders for QoL, exercise capacity, echocardiographic response, and global response across ARNI dose categories (100, 200, and 400 mg/day) and stratified by ischemic versus non-ischemic etiology. Percentages are shown together with absolute sample sizes (*n*) within each cell. This figure is descriptive and intended for exploratory visualization. Cells with small sample sizes (*n* ≤ 5) should be interpreted with caution. Figure S2. Estimated probability of echocardiographic response according to CRT duration. The probability of being classified as an echocardiographic responder (ΔLVEF ≥ 5%) is shown as a function of CRT duration prior to ARNI initiation, modeled using logistic regression with cubic spline functions. The solid line represents the estimated probability and the shaded area the 95% confidence interval. The dashed vertical line indicates the approximate transition zone around 66 months (5.5 years). Predictions are displayed only within the range of CRT duration supported by the observed data. Individual patient observations are indicated along the x-axis.

## Abbreviations

The following abbreviations are used in this manuscript:

ARNI	Angiotensin Receptor–Neprilysin Inhibitor
CRT	Cardiac Resynchronization Therapy
HFrEF	Heart Failure with Reduced Ejection Fraction
GDMT	Guideline-Directed Medical Therapy
T0	Baseline evaluation
T1	Follow-up evaluation
KCCQ-12	12-item Kansas City Cardiomyopathy Questionnaire
QoL	Quality of Life
NYHA	New York Heart Association
LVEF	Left Ventricular Ejection Fraction
LV	Left Ventricle
MAPSE	Mitral Annular Plane Systolic Excursion
CRT-P	Cardiac Resynchronization Therapy–Pacemaker
CRT-D	Cardiac Resynchronization Therapy–Defibrillator
ACE	Angiotensin-Converting Enzyme
ARB	Angiotensin Receptor Blocker
ESC	European Society of Cardiology
HFA	Heart Failure Association
ECG	Electrocardiogram
LVEDV	Left Ventricular End-Diastolic Volume
LVESV	Left Ventricular End-Systolic Volume
LAV	Left Atrial Volume
ICD	Implantable Cardioverter-Defibrillator
VT	Ventricular Tachycardia
AF	Atrial Fibrillation
NT-proBNP	N-terminal pro–B-type Natriuretic Peptide
