# Supplementary material for: Impact of Late ARNI Initiation on Quality of Life and Functional Capacity in CRT-Treated HFrEF Patients: A Single-Centre Cohort Study
Source: J Clin Med. 2026 Feb 19;15(4):1617. doi: 10.3390/jcm15041617 (PMC12941977; doi:10.3390/jcm15041617)
Supplement: Supplementary file 1 [file jcm-15-01617-s001.zip › jcm-4133932-supplementary.pdf]

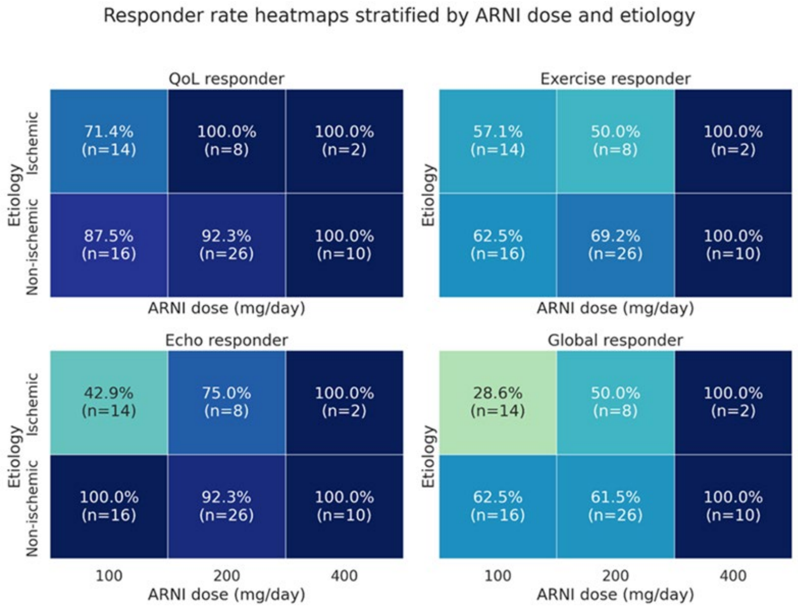

Figure S1. Responder rate heatmaps stratified by ARNI dose and cardiomyopathy etiology. Heatmaps display the proportion of responders for QoL, exercise capacity, echocardiographic response, and global response across ARNI dose categories (100, 200, and 400 mg/day) and stratified by ischemic versus non-ischemic etiology. Percentages are shown together with absolute sample sizes (n) within each cell. This figure is descriptive and intended for exploratory visualization. Cells with small sample sizes (n ≤ 5) should be interpreted with caution.

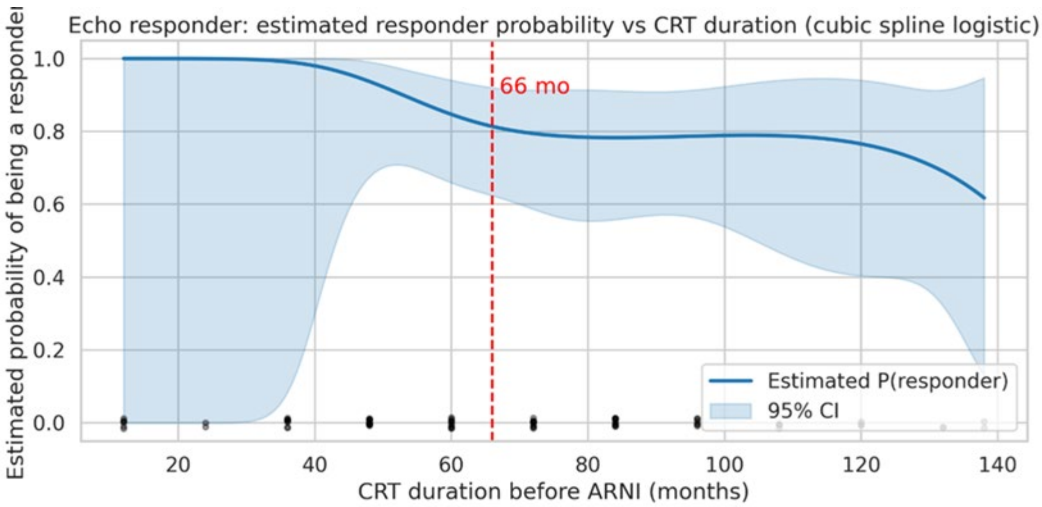

Figure S2. Estimated probability of echocardiographic response according to CRT duration. The probability of being classified as an echocardiographic responder (ΔLVEF ≥ 5%) is shown as a function of CRT duration prior to ARNI initiation, modeled using logistic regression with cubic spline functions. The solid line represents the estimated probability and the shaded area the 95% confidence interval. The dashed vertical line indicates the approximate transition zone around 66 months (5.5 years). Predictions are displayed only within the range of CRT duration supported by the observed data. Individual patient observations are indicated along the x-axis.
